# A Case of Granulomatosis with Polyangiitis (Wegener's Granulomatosis) Presenting with Marked Inflamed Tracheobronchial Mucosa

**DOI:** 10.1155/2013/208194

**Published:** 2013-09-30

**Authors:** Teruaki Nishiuma, Hisashi Ohnishi, Sho Yoshimura, Saori Kinami, Susumu Sakamoto

**Affiliations:** ^1^Department of Respiratory Medicine, Akashi Medical Center, 743-33 Yagi, Okubo-cho, Akashi, Hyogo 674-0063, Japan; ^2^Department of Respiratory Medicine, Kakogawa West City Hospital, 384-1 Hiratsu, Yoneda-cho, Kakogawa, Hyogo 675-8611, Japan; ^3^Department of Internal Medicine, Akashi Medical Center, 743-33 Yagi, Okubo-cho, Akashi, Hyogo 674-0063, Japan

## Abstract

A 70-year-old man was admitted to our hospital because of weight loss and persistent dry cough. Chest radiograph and CT showed multiple infiltrates in the bilateral upper lobes and the remarkably thickened bronchial walls. Bronchoscopy revealed diffuse erythema and edema of the tracheobronchial mucosa without any ulcerous legions. Serum MPO-ANCA was positive (155 EU). Transbronchial biopsy was performed and revealed necrotic granulomas with multinucleated giant cells in the bronchial/bronchiolar and parenchymal lesions. Thus, we diagnosed it as a localized form of granulomatosis with polyangiitis (GPA, Wegener's granulomatosis). After treatment with corticosteroid and cyclophosphamide, the bronchial findings were entirely resolved. We report here a rare case of GPA presenting with markedly inflamed tracheobronchial mucosa.

## 1. Introduction

Granulomatosis with polyangiitis (GPA, Wegener's granulomatosis) is one of the systematic vasculitides involved in various organs such as the upper respiratory tract, the lungs, and the kidneys, characterized pathologically by necrotizing granulomatous inflammation [1, 2]. Although the pulmonary involvement of GPA is well described, the lower airway findings are not frequently remarked, and various descriptions have been reported [1, 3]. The most common airway abnormality in GPA consists of mucosal edema, erythema, thickening, and granularity of mucosal surface [1]. In this paper, we present a case of GPA with markedly inflamed bronchial mucosa that was characteristically found in chest CT scan and bronchoscopic examination.

## 2. Case Report

**Figure 1:**
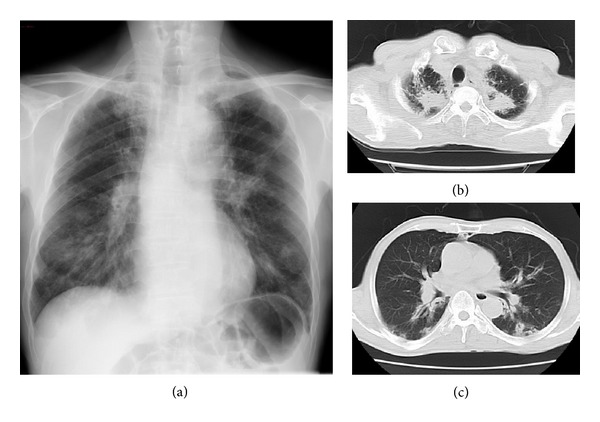
(a) Chest X-ray showed multiple small infiltrates in both lungs. ((b), (c)) Chest CT showed multiple nodules in the apex and the remarkable bronchial wall thickening in lower areas of both lungs.

A 70-year-old man was admitted to our hospital because of weight loss, general fatigue, and abnormal findings of the chest radiographs. He had a smoking history of 20 pack-year and suffered from cerebral infarction twice (2 and 25 years ago). Three months before admission, he developed occasional dry cough, nasal bleeding, taste disturbance, and refractory otitis media. He did not notice high fever more than 38.0°C. His primary physician did not detect any abnormal findings in the chest radiographs at that time. Two months later, he consulted the doctor again due to his persistent cough and approximately 10 Kg weight loss within the last two months. Since the chest radiographs showed multiple small infiltrates in both lungs (Figure 1), he was admitted to our hospital.

On examination, the height was 155 cm and the weight was 55.3 kg. The temperature was 37.0°C, the blood pressure was 130/80 mmHg, the pulse was 110 beats per minute, and the oxygen saturation was 93% while he was breathing ambient air. His lungs were clear to auscultation bilaterally. His abdomen was neither tender nor distended. Saddle-nose deformity was not seen. There was no edema, eruption, or clubbing of his extremities. The other physical examination was not notable. Laboratory studies showed the increase of white blood cell counts and C-reactive protein (Table 1). Proteinuria and hematuria were not detected, and urinary sediment was normal. Rheumatoid factor was 265 U/mL, and the antinuclear antibody was negative. MPO-ANCA was raised (155 IU/L), whereas PR3-ANCA was negative. Serum sIL-2 receptor was also increased (2220 U/mL). Pulmonary function test was within normal limits. Thin section of computed tomography (CT) scan revealed multiple infiltrates in the upper lobes and the remarkably thickened bronchial wall and consolidation around the bronchi in lower lobes (Figure 1). Upon examination by otolaryngologist, his nasal mucosa was intact, and his paranasal sinus CT showed no abnormal findings; therefore, nasal biopsy was not performed. 

**Figure 2:**
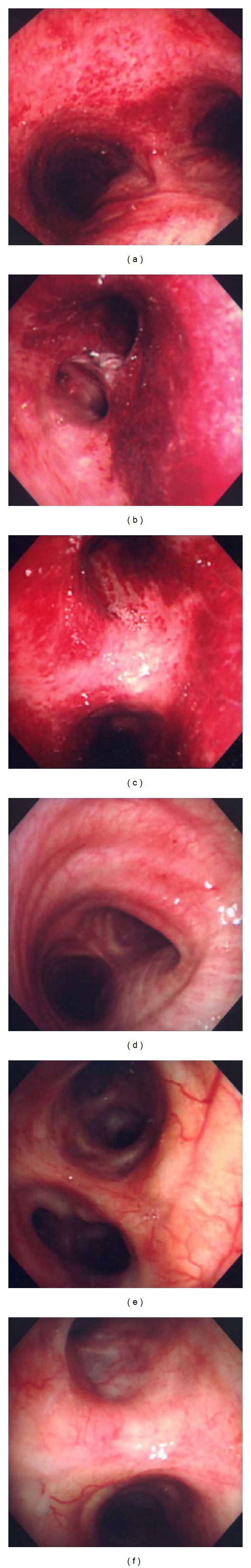
Bronchoscopic findings revealed that almost all bronchial walls were covered with diffuse vessels-rich erythema and edema without any ulcer on the fifth day ((a)–(c)) and two months after hospital admission ((d)–(f)). Representative images of the carina ((a), (d)), the left main bronchus ((b), (e)), and the second carina of the right lung ((c), (f)) are shown.

**Figure 3:**
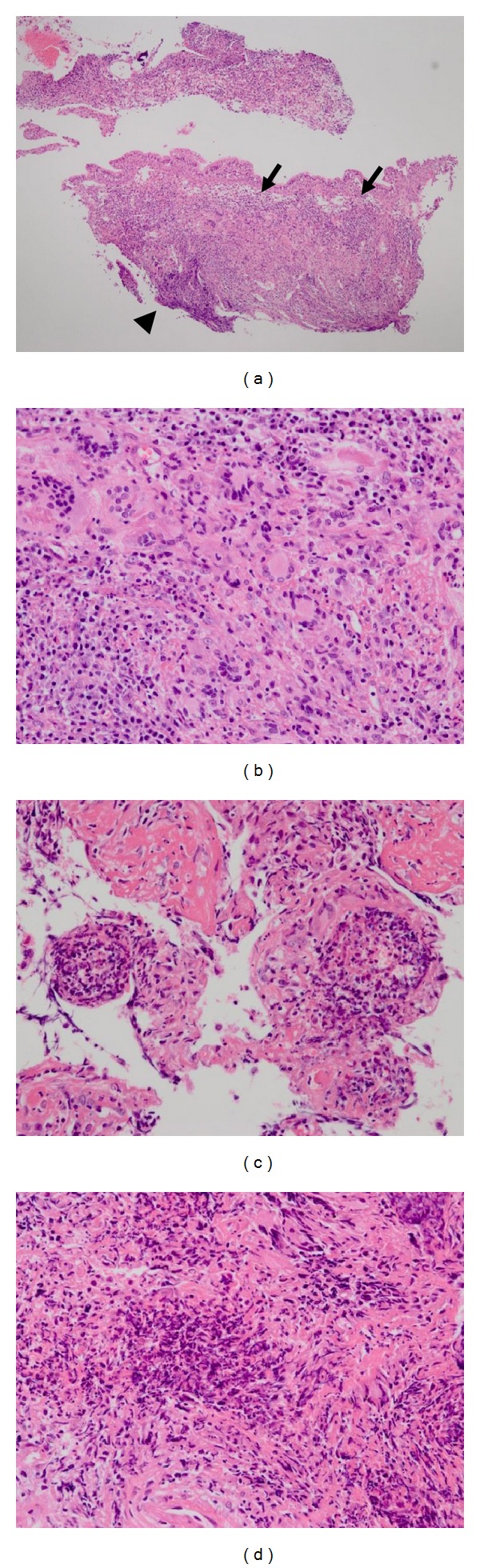
Histologic findings from the transbronchial biopsy specimens obtained from the right second carina ((a), (b)) and the nodule on the apex of the right lung ((c), (d)) using hematoxylin and eosin stains. Granuloma formation accompanied by abundant inflammatory cells ((a), *arrow*) and many multinucleated giant cells was found in the right second carina, surrounded by neutrophil-infiltrated microabscess on bronchial mucosa ((a), *arrow head*). The histology of the nodules on the right upper lobe also showed inflammatory cells including nodular necrotizing foci (c) and multinucleated giant cells (d). Obvious vasculitis was not found.

On the fifth day, bronchoscopy was performed which revealed diffuse erythema and edema of the tracheobronchial mucosa (Figure 2). These changes were located throughout the region between the carina and the visible peripheral bronchi without any contact bleeding. Then, bronchial alveolar lavage (BAL) was performed from the right middle lobe. BAL fluid analysis showed the small increase of neutrophils (total cell counts: 280/*μ*L, neutrophils: 18.6%, macrophages: 75.0%, lymphocytes: 6.4%, eosinophils: 0.0%) and no growth of bacterial culture. Lymphocytic surface marker in BAL fluid showed no specific findings suggesting lymphoproliferative diseases. Histological examination of the right second carina showed granuloma formation accompanied by abundant inflammatory cells (Figure 3(a), *arrow*) and many multinucleated giant cells (Figures 3(a) and 3(b)), surrounded by neutrophil-infiltrated microabscess on bronchial mucosa (Figure 3(a), *arrow head*). Transbronchial biopsy of nodules on the right upper lobe showed inflammatory cells including nodular necrotizing foci (Figure 3(c)) and multinucleated giant cells (Figure 3(d)). Although the obvious vasculitis was not found, we diagnosed this as GPA on the basis of these histopathological findings and positive MPO-ANCA.

 We then treated the patient with intravenous methylprednisolone pulse therapy (1 g/day) for three days followed by 50 mg of oral prednisolone and 100 mg of cyclophosphamide. The symptoms and the chest X-ray findings were rapidly restored. After two months of treatment, the level of MPO-ANCA became normal, and the affected bronchial mucosa was completely improved. Drugs were gradually decreased to only 10 mg of prednisolone without recurrence. Four years later, the patient died because of aspiration pneumonia without recurrence of GPA.

## 3. Discussion

Granulomatosis with polyangiitis (GPA) is characterized by necrotizing granulomatous inflammation of upper and lower respiratory tracts, glomerulonephritis, and necrotizing vasculitis of the lungs and a variety of systemic organs and tissues. Lung parenchymal disease is the most frequent manifestation which produces multiple nodules and masses. On the other hand, airway involvement in GPA does not show typical radiographic pattern. In large airways, these findings may consist of focal or elongated segments of bronchial stenosis and intraluminal soft tissue mass or bronchial thickening with or without lobar or segmental atelectasis [4]. Bronchiectasis and peribronchial thickening in small airways were reported in approximately 40% of GPA [5].

Bronchoscopic examination is thought to be useful in detecting the abnormal bronchial findings of GPA compared with CT scan. It is reported that airway involvement is observed in 15%–55% of patients with GPA [1, 6]. There are few large studies about endoscopic findings of GPA by bronchoscopic examination. For example, Cordier et al. [3] reported that 41 (55%) of 74 patients who underwent bronchoscopy showed abnormal findings, which consisted of bronchial stenosis (13 patients), ulcerations or pseudotumor (7 patients), inflammatory lesions without stenosis (10 patients), isolated hemorrhage (10 patients), and isolated purulent secretions (1 patient). They found no striking differences between the localized GPA and the other classical GPA. Daum et al. [6] also reported that of 51 patients with biopsy-proven GPA who underwent bronchoscopy, 30 (59%) had endobronchial abnormal findings: subglottic stenosis was shown in 5 patients, ulcerative tracheobronchitis in 18 patients, tracheal or bronchial stenosis in 4 patients, and hemorrhage without identifiable source in 2 patients. Unlike these findings, our case showed diffuse vessels-rich erythema and edema without ulcerative lesion or erosion. Interestingly, the pulmonary function test was normal, regardless of the notably widespread findings by the endoscopic examination and the bronchial wall thickening by the chest CT scan. Pauls et al. [7] reported a similar rare case with isolated thickening of the bronchial walls with peribronchiolar consolidations in the CT scan. Serum PR3-ANCA was positive, and they documented ulcerative bronchitis. 

Histologically, we diagnosed this as GPA from the transbronchial biopsy specimens, although lung biopsy usually needs to be performed through open or thoracoscopic biopsy. For the definition of GPA, microscopic polyangiitis (MPA) was distinguished from GPA and eosinophilic granulomatosis with polyangiitis (EGPA, Churg Strauss syndrome) by the absence of granuloma formation and the presence of a necrotizing vasculitis [8, 9]. It is known that tissue samples from upper and lower respiratory tracts are small and sometimes do not allow the definitive identification of vasculitis [10]. Biopsies of tracheal and bronchial masses often show fibrinoid necrosis, microabscess, and infiltration by epithelioid histiocytes and neutrophils without vasculitis [11]. Meanwhile, in the acute lesions of GPA, the prominent neutrophil inflammation has the appearance of an abscess more than a granuloma [12]. In the chronic phase, the pattern of injury shows an irregular central zone of necrosis containing varying numbers of degenerating neutrophils and necrotic debris surrounded by poorly defined granulomatous inflammation with palisades of elongated macrophages and scattered multinucleated giant cells [12]. Although we did not detect the definitive evidence of vasculitis, histopathological findings showed granulomatous inflammation with many multinucleated giant cells, surrounded by neutrophilic microabscess on bronchial mucosa. Since clinical manifestations and several examinations showed no affected organs other than the upper respiratory tract and lungs, we diagnosed it as a localized form of GPA, according to EUVAS (European Vasculitis Study) disease categorization of ANCA-associated vasculitis [13]. 

Granuloma formation is a key pathologic finding in two of the ANCA-associated vasculitides: GPA and EGPA [14]. It is suggested that PR3 (and possibly also MPO) displays features of an endogenous “danger signal” inducing autoinflammation [14]. Although the diagnostic sensitivity and specificity of PR3-ANCA and MPO-ANCA for the ANCA-associated vasculitis are very high, a minority of patients with GPA has MPO-ANCA, which indicates the diagnosis of MPA. It is known that PR3-ANCA was found in 70% to 90% of patients with active GPA, and MPO-ANCA was observed in only 5% to 10% of patients with GPA [15, 16]. The racial difference may affect the selection of PR3-ANCA and MPO-ANCA in the incident of vasculitis. Fujimoto et al. showed that MPA and MPO-ANCA were the predominant subtypes in Japan, while GPA and PR3-ANCA were predominant in the UK [17]. Chen et al. also reported that patients with MPO-ANCA positive GPA were not rare in Chinese subjects [15]. However, it is reported that MPO-ANCA positive GPA patients showed less organ involvement than PR3-ANCA positive GPA patients [16].

Tracheobronchial involvement with GPA has several manifestations, including tracheal and bronchial stenosis, mass lesions, tracheobronchial malacia, and tracheoesophageal fistulae [1]. Subglottic and tracheal stenosis is frequently reported in many articles; however, bronchial ulceration and stenosis are less common than tracheal stenosis. Since this patient had neither ulcer nor stenosis in the bronchi, he had no severe respiratory symptoms in spite of severe bronchial inflammation. The combination therapy with prednisolone and cyclophosphamide completely improved the bronchial change. 

## Figures and Tables

**Table 1:** Laboratory data on admission.

Hematology	
WBC	14750/*μ*L
Neu	93.4%
Lym	4.7%
Mono	1.7%
Eos	0.2%
Baso	0.1%
RBC	374 × 10^4^/*μ*L
Hb	11.3 g/dL
Ht	34.0%
PLT	50.2 × 10^4^/*μ*L
Biochemistry	
TP	5.8 g/dL
ALB	2.1 g/dL
AST	52 IU/L
ALT	50 IU/L
LDH	218 IU/L
BUN	17.5 mg/dL
CRE	0.84 mg/dL
Na	137 mEq/L
K	4.3 mEq/L
CL	101 mEq/L
Serology	
CRP	17.5 mg/dL
ESR	124 mm/hr
IgG	1540 mg/dL
IgA	326 mg/dL
IgM	62 mg/dL
ACE	8.0 U/L
ANA	<40
RF	265 U/mL
sIL-2R	2220 U/mL
PR3-ANCA	(—)
MPO-ANCA	155 EU
*β*-D glucan	<5.0 pg/mL
*Aspergillus* Ab	(—)
*Cryptococcus* Ag	(—)
Urinalysis	
Protein	(—)
Glucose	(—)
Occult blood	(—)
Urinary sediment	Normal

## References

[1] Polychronopouls VS, Prakash UBS, Golbin JM (2007). Airway involvement in Wegener's granulomatosis. *Rheumatic Disease Clinics of North America*.

[2] Jennette JC, Falk RJ, Bacon PA (2013). 2012 revised International Chapel Hill Consensus Conference Nomenclature of Vasculitides. *Arthritis and Rheumatism*.

[3] Cordier J-F, Valeyre D, Guillevin L, Loire R, Brechot J-M (1990). Pulmonary Wegener’s granulomatosis. A clinical and imaging study of 77 cases. *Chest*.

[4] Lee KS, Kim TS, Fujimoto K (2003). Thoracic manifestation of Wegener’s granulomatosis: CT findings in 30 patients. *European Radiology*.

[5] Screaton NJ, Sivasothy P, Flower CDR, Lockwood CM (1998). Tracheal involvement in Wegener’s granulomatosis: evaluation using spiral CT. *Clinical Radiology*.

[6] Daum TE, Specks U, Colby TV (1995). Tracheobronchial involvement in Wegener’s granulomatosis. *The American Journal of Respiratory and Critical Care Medicine*.

[7] Pauls S, Krüger S, Barth TF, Brambs H-J, Juchems MS (2007). Atypical bronchial thickening and ulceration: a rare radiological finding in Wegener’s granulomatosis. *The British journal of radiology*.

[8] Jennette JC, Falk RJ, Andrassy K (1994). Nomenclature of systemic vasculitides: proposal of an international consensus conference. *Arthritis and Rheumatism*.

[9] Watts R, Lane S, Hanslik T (2007). Development and validation of a consensus methodology for the classification of the ANCA-associated vasculitides and polyarteritis nodosa for epidemiological studies. *Annals of the Rheumatic Diseases*.

[10] Schnabel A, Holl-Ulrich K, Dalhoff K, Reuter M, Gross WL (1997). Efficacy of transbronchial biopsy in pulmonary vaculitides. *European Respiratory Journal*.

[11] Yilmaz A, Damadoğlu E, Aksoy F, Düzgün S, Yağci Tuncer L, Yalçinsoy M (2006). A relapsing case of Wegener’s granulomatosis presenting as an endobronchial mass. *Tuberkuloz ve Toraks*.

[12] Mukhtyar C, Guillevin L, Cid MC (2009). EULAR recommendations for the management of primary small and medium vessel vasculitis. *Annals of the Rheumatic Diseases*.

[13] Jennette JC (2011). Nomenclature and classification of vasculitis: Lessons learned from granulomatosis with polyangiitis (Wegener’s granulomatosis). *Clinical and Experimental Immunology*.

[14] Lamprecht P, Wieczorek S, Epplen JT, Ambrosch P, Kallenberg CGM (2009). Granuloma formation in ANCA-associated vasculitides. *APMIS*.

[15] Chen M, Yu F, Zhang Y, Zou W-Z, Zhao M-H, Wang H-Y (2005). Characteristics of Chinese patients with Wegener’s granulomatosis with anti-myeloperoxidase autoantibodies. *Kidney International*.

[16] Schönermarck U, Lamprecht P, Csernok E, Gross WL (2001). Prevalence and spectrum of rheumatic diseases associated with proteinase 3-antineutrophil cytoplasmic antibodies (ANCA) and myeloperoxidase-ANCA. *Rheumatology*.

[17] Fujimoto S, Watts RA, Kobayashi S (2011). Comparison of the epidemiology of anti-neutrophil cytoplasmic antibody-associated vasculitis between Japan and the UK. *Rheumatology*.

